# 

**Published:** 1912-01-06

**Authors:** 


### Florence Nightingale Memorial.

We have to acknowledge the receipt of the following contributions since last week: B. Lazarus (Rhodesia), 2s. 6d.; received by Mr. G. Q. Roberts: The Worshipful Company of Skinners, £21; the Earl of Radnor, £10 10s.; collection at Wellington College Sanatorium, £5 5s.; F. W. Darby, Esq., £5 5s.; Mrs. Richardson, £5; Miss Stancomb-Wills, £5; Mrs. Kingsley, £5; collection at Hillside Homes, Perth, £4; Queen's Nurses (English Branch) (additional), £3 3s.; Major Robert Gordon, D.S.O., £2 2s.; Mrs. Proctor, £2; Miss Emily Dixon, £1 6s. 6d.; Surg.-Gen. Sir Lionel Spencer, K.C.B., K.H.S., £1 1s.; Field-Marshal Sir Evelyn Wood, G.C.B., G.C.M.G., V.C., £1 1s.; A. St. G. Hamersley, Esq., K.C., M.P., £1 1s.; Sir Thomas Rees Price, K.C.M.G., £1 1s.; Thomas Hughes, Esq., £1 1s.; Mrs. L. E. Opdycke, £1; Miss A. Jones, Matron Borough Hospital, Carnarvon, 10s. 6d.; Miss M. N., Oxford, 10s. 6d.; Miss A. Wood (annuities), 9s.; Miss H. M. Basham, 5s.; Women's Co-operative Guild (Stratford Branch), 4s. 3d.; the children of Atherstone School, Warwickshire, 2s. 6d.; Miss E. V. Yeatman, 2s. 6d.; Anon., 2s. 6d.

It is requested that all those who have taken lists or are making collections will send in their results not later than January 14, as it is desired to close the Fund on or about that date. All contributions should be sent before January 14 to the Editor, The Hospital, 29 Southampton Street, Strand, London, W.C., or to the Hon. Sec. Florence Nightingale Memorial, St. Thomas's Hospital, Albert Embankment, London, S.E.

### Appointments.

The following reappointments have been made at the Manchester Royal Infirmary: Mr. G. E. Loveday, M.B., B.C. Cantab., M.R.C.S., L.R.C.P., Director of the Clinical Laboratory; Mr. A. E. Barcley, M.A., B.C. Cantab., M.R.C.S., L.R.C.P., Medical Officer for Radiography and Electricity Department; Mr. S. R. Wilson, M.B., B.S. Lond., F.R.C.S. Edin., Second Anæsthetist; and Mr. T. Coogan, M.B., Ch.B. Vict., Fifth Anæsthetist.

Mr. H. St. Clair Roberts has been appointed Hon. Surgeon in the place of Mr. C. Pollard, F.R.C.S., resigned, at the Worcester Ophthalmic Hospital.

Dr. Frank Webb, Newcastle-under-Lyme, has been appointed Medical Officer for Leigh in succession to Dr. Wynne, who has been appointed Medical Officer of Wigan.

Mr. Charles E. Price, M.R.C.S., L.R.C.P., has been appointed Medical Officer to Bromley (Kent) Union Workhouse and Infirmary.

